# Genetic Differentiation in Anthocyanin Content among Berry Fruits

**DOI:** 10.3390/cimb43010004

**Published:** 2021-04-29

**Authors:** Alicja Ponder, Ewelina Hallmann, Martyna Kwolek, Dominika Średnicka-Tober, Renata Kazimierczak

**Affiliations:** Department of Functional and Organic Food, Institute of Human Nutrition Sciences, Warsaw University of Life Sciences, Nowoursynowska 159c, 02-776 Warsaw, Poland; alicja_ponder1@sggw.edu.pl (A.P.); martyna.d.kwolek@gmail.com (M.K.); dominika_srednicka_tober@sggw.edu.pl (D.Ś.-T.); renata_kazimierczak@sggw.edu.pl (R.K.)

**Keywords:** anthocyanin, bioactive compounds, HPLC method, berry fruits, raspberry, blackberry, red currant, blackcurrant, highbush blueberry

## Abstract

Anthocyanins are widely distributed secondary metabolites that play an essential role in skin pigmentation of many plant organs and microorganisms. Anthocyanins have been associated with a wide range of biological and pharmacological properties. They are also effective agents in the prevention and treatment of many chronic diseases. Berries are particularly abundant in these compounds; therefore, their dietary intake has health-promoting effects. The aim of this study was to identify and determine the anthocyanin content in selected species and cultivars of berry fruits, such as raspberry, blackberry, red currant, blackcurrant, and highbush blueberry, widely consumed by Europeans. The concentrations of anthocyanins were determined by HPLC, identifying individual compounds: cyanidin-3-*O*-glucoside, pelargonidin-3-*O*-glucoside, delphinidin-3-*O*-glucoside, delphinidin-3-*O*-rutinoside, cyanidin-3-*O*-rutinoside, delphinidin-3-*O*-galactoside, cyanidin-3-*O*-galactoside, and malvidin-3-*O*-galactoside. The experimental data showed that the selected species and cultivars of berry fruits differ significantly in the contents of anthocyanins. Among all species tested, blackberry and blackcurrant were characterized significantly by the highest content of anthocyanins (sum), while the lowest content was found in red currant fruits. Additionally, the content of individual anthocyanin compounds in particular species and cultivars was also different. Considering the high content of anthocyanins and their potential positive impact on human health and protection against disease, berries should be part of healthy nutrition.

## 1. Introduction

**Figure 1:**
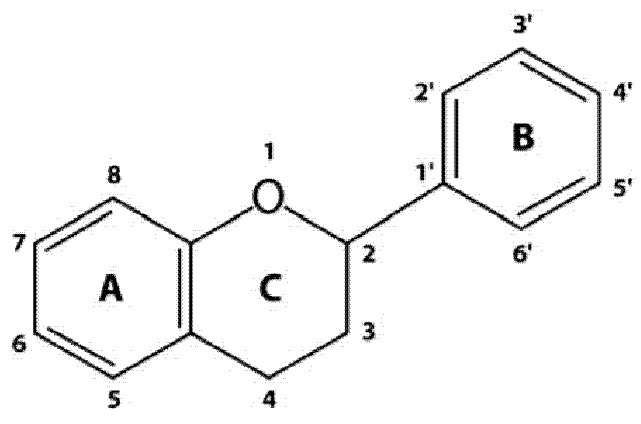
Diphenylpropane skeleton (C_6_C_3_C_6_) of all flavonoids.

Anthocyanins are widely distributed secondary metabolites that play an essential role in skin pigmentation of many plant organs and microorganisms [1]. They are flavonoids by classification, sharing the same diphenylpropane skeleton (C_6_C_3_C_6_) (Figure 1), and are predominantly found in berry fruits [2]. Actually, 25 different anthocyanidins are known, which differ from each other in the presence of hydroxyl (−OH) and methoxy (−OCH3) groups bound at the scaffold core [3]. These compounds have an important function for the quality control of foods that claim to contain specific anthocyanin-enriched fruits. The characteristic anthocyanin pattern is specific for each fruit. Based on this fingerprinting, it is possible to recognize the adulteration and/or sophistication of particular products that claim to contain a certain fruit. This method allows for the detection of a misidentification of the initial plant raw material or adulteration with other plants [3,4,5,6]. 

Anthocyanins are water soluble, and their spectral properties are usually responsible for the red, blue, and purple coloring of different plant parts (flowers, fruits, and other plant tissues). This is due to the accumulation of anthocyanins in the flesh and skin of the fruit [7,8]. In the case of fruits like peaches, apricots, plums, and grape berries, anthocyanins accumulate more in the skin than in the flesh [1]. Their specific color depends on the environmental pH and the presence, at the same time, of other compounds from flavone and flavonol groups and metal ions [9]. Depending on the pH value, anthocyanins can exist in many different protonated, deprotonated, hydrated, and isomeric forms. Due to changes in protonation and hydration at acidic pH (pH 1–3), anthocyanins are red; at pH 4–5, they are colorless or yellow; and at pH values between 6 and 7, they became blue-purple [10,11,12]. The role of anthocyanins in plants is in attracting plant pollinators and increasing stress tolerance. Floral color and scent are the traits that attract pollinators. Anthocyanins provide flowers with radiance and attractiveness, which can act as a beneficial visualization signal to pollinators, increasing the number of pollinator visits and thereby increasing the pollinator foraging efficiency and reproductive success. Additionally, apart from the floral parts, anthocyanins often occur transiently in the root, stem, fruits, and leaf tissues of plants at specific developmental stages in response to unfavorable environmental factors, including UV radiation, cold temperatures, and water stress. They play an important role in the natural protective mechanism of plants [3,13].

Moreover, fruits, especially berries, are particularly abundant in different bioactive compounds, in that anthocyanins. The beneficial effects of these compounds for human health have been known from at least the 16th century [10]. There are a great number of studies devoted to the different biological effects exerted by anthocyanins. Anthocyanins have been associated with a wide range of biological and pharmacological properties. They are also effective in the prevention and treatment of many chronic diseases [11,12,13,14]. They exhibit antioxidant activity and inactivating properties against free radicals; moreover, anthocyanins can provide anti-inflammatory, antiviral, and anticancer effects [14]. The anticancer activity of anthocyanins contained in food results from their properties consisting of binding of mutagens, inhibiting metabolic activation of pro-mutagens, removing free radicals, inhibiting cancer cell proliferation, intensifying DNA repair processes, and inducing apoptosis of cancer cells. They can also influence cell differentiation, exhibit cytotoxic and cytostatic effects against cancer, and regulate the immune system [14].

Nevertheless, anthocyanins are also used in the development of food colorants and functional ingredients in food. However, the use of anthocyanins as food colorants and functional foods ingredients is limited because of the low stability of anthocyanins under the environmental conditions (heat, oxygen, and light among others) experienced during processing and storage [11,15,16]. The anthocyanin content depends on the balance between biosynthesis and degradation. Genetic, developmental, and environmental factors all regulate anthocyanin metabolism. The genetic differences found in raw materials appear to be highly relevant for the anthocyanin content [17]. This study will be helpful in designing strategies for obtaining anthocyanin-rich crops via species and variety control.

Anthocyanin compounds form noncovalent complexes with other flavonoids (co-pigments), such as flavones and flavonols, that can stabilize the color. This phenomenon is called co-pigmentation. The co-pigmentation complex is, however, more stable when the anthocyanin pigments are acylated, as the acylated pigments form more stable complexes when they are linked through the sugar residue by aromatic and/or aliphatic phenolic acyl moieties. Therefore, acylated anthocyanins have improved color stability in the 4–5 pH range and retain the color in the mildly acidic pH environment of many food products as compared to nonacylated anthocyanins, which are nearly colorless at this pH range. Acylated anthocyanins can also withstand degradation at higher temperatures and at longer light exposures. As a result, foods added with acylated anthocyanin colorants have a longer shelf-life [18].

Berry-type fruits have long been regarded as having considerable health benefits because of their nutritional attributes, particularly their total antioxidant activity against cellular oxidation reactions. These discoveries have stimulated research to investigate the anthocyanins status of distinct berry fruit species and cultivars from different countries. There are a great variety of species from diverse botanical families (e.g., raspberry (*Rubus ideus* L.), blackberry (*Rubus* L.), red currant (*Ribes* L.), blackcurrant (*Ribes nigrum* L.), highbush blueberry (*Vaccinium corymbosum* L.) that produce the small purple or red fruits that are denoted as berries. In botanical terms, a berry is a fruit with many seeds and mesocarp flesh that evolves from a flower with a superior ovary [11,19]. Increased consumption of berry fruits, which are rich in nutrients and bioactive compounds like anthocyanins, can prevent various diseases and disorders. Bioactive compounds from berries have potent antioxidant, anticancer, antimutagenic, antimicrobial, anti-inflammatory, and antineurodegenerative properties [20,21,22,23,24,25]. Berry bioactive components impart anticancer effects through various complementary and overlapping mechanisms of action, including the induction of metabolizing enzymes, modulation of gene expression, and their effects on cell proliferation, apoptosis, and subcellular signaling pathways. Anthocyanins have received considerable interest in bearing possible relations to human health [20,26,27,28]. Along with fresh berries, a variety of berry products such as juice, wine, jam, and food colorants contribute significantly to the intake of anthocyanins. Additionally, there has been a growing trend in the use of berry extracts as ingredients in functional foods and dietary supplements [20]. 

Nowadays, a large number of scientific studies have established that dietary intake of berry fruits has a positive and profound effect on human health, performance, and disease protection, which are related to the high anthocyanin content of the berries. Therefore, in this paper, the identification and determination of the anthocyanin content in selected species and cultivars of berry fruit (raspberry (*Rubus ideus* L. Tulnameen cv.), blackberry (*Rubus* L. Darrow and Thornless Evergreen cv.), red currant (*Ribes* L. Detvan, Rovada, Heros, and Jonkheer van Tets cv.), blackcurrant (*Ribes nigrum* L. Ben Lomond and Titania cv.), and highbush blueberry (*Vaccinium corymbosum* L., Coville, Blue Gold, and Brigitta cv.)) were performed. Given the wide consumption of berry fruits and their potential positive impact on human health and disease protection, scientific research like this is important and useful. Moreover, it is equally important to disseminate this information to the general public in order to raise awareness of the beneficial effects of anthocyanin-rich products.

## 2. Materials and Methods

### 2.1. Chemicals and Reagents

The following chemicals and reagents were obtained: acetonitrile 99.9% (HPLC grade, CAS no. 75-05-8), methanol (HPLC grade, CAS no. 67-56-1), acetic acid (glacial grade, CAS no. 64-19-7), and water (HPLC grade, CAS no. 7732-18-5). In addition, the following anthocyanin standards were obtained: cyanidin-3-*O*-glucoside (CAS no. 7084-24-4), pelar-gonidin-3-*O*-glucoside (CAS no. 18466-51-8), delphinidin-3-*O*-glucoside (CAS no. 6906-38-3), delphinidin-3-*O*-rutinoside (CAS no. 15674-58-5), cyanidin-3-*O*-rutinoside (CAS no. 18719-76-1), delphinidin-3-*O*-galactoside (CAS no. 27661-36-5), cya-nidin-3-*O*-galactoside, (CAS no. 27661-36-5), and malvidin-3-*O*-galactoside (CAS no. 643-84-5). All chemicals and reagents are produced by Sigma-Aldrich Chemical Company (Warsaw, Poland).

### 2.2. Plant Material Origin

Different species of berry fruits were collected directly from producers at the full maturity stage (characteristic for every species and cultivar). Farms were located in the Mazovia region in Poland. Experimental farms belonged to the organic farming system. According to law, the owners had qualified plant material as well a certificate validated by plant material producers on their farm. The mean amount of berry sample was 1 kg per cultivar. Fruit samples were transported to the laboratory and immediately separated into two parts; one part was used for dry matter determination and the other part for freeze-drying under the following conditions: temperature −50 °C, pressure 0.100 mBar, time 72 h, and Freeze-drier LabconCo 2.5 equipment (Poland). After the drying process, fruits were milled with the A-80 laboratory mill.

### 2.3. Dry Matter Determination

The dry matter content was determined using the weight method. A small glass beaker was weighed and the mass was written down. Next, fresh berry fruits were transferred into the beaker. The mass was weighed again. Samples were put into a laboratory drier (condition 105 °C) and left there for 48 h. After that time, the samples were transferred into a desiccator and left for the next 12 h. The samples were weighed, and the total dry matter was calculated on the basis of mass differentiation, as described by Ohene and Maalekuu [29].

### 2.4. Anthocyanin Separation and Identification

Anthocyanins were measured by the HPLC method described by Ponder and Hallmann [30]. Freeze-dried berry fruit samples were extracted with 80% methanol. After first centrifugation (conditions: time 10 min, rpm 6000, temperature 30 °C), 2.5 mL of the supernatant was collected in a new plastic tube, and then 2.5 mL of 10 mol HCl and 5 mL of 100% methanol were added. The samples were gently shaken (up and down) and put in a cold place (5 °C, 10 min). Next, 900 µL of the extract was transferred into HPLC vials and analyzed. The anthocyanins were separated under isocratic conditions with a flow rate of 1.5 mL/min. One mobile phase, 5% acetic acid, methanol, and acetonitrile (70:10:20), was used. The HPLC set was performed from modules: two pumps (LC-20AD), one controller (CBM-20A), one column oven (SIL-20AC), one spectrometer (UV–VIS SPD-20 AV). Phenomenx Fusion 80-A (4.6 × 250 mm, practical shape 4 µm), and one column (C18) were used. The analysis time was 15 min at a wavelength of 520 nm. The anthocyanins were identified by using pure standards (Sigma-Aldrich, Poland) and the retention times for the standards. The limit of detection (LOD) and level of quantification (LOQ) for all quantified anthocyanin compounds are presented below (Table 1).

### 2.5. Statistical Analysis

The obtained results were statistically elaborated using Statgraphics Centurion 15.2.11.0 software (StatPoint Technologies, Inc., Warranton, VA, USA). PCA was performed with XLStat Trial version (Microsoft Excel). In tables, values represent the mean value for berry fruit cultivars: Tulnamen raspberry (*n* = 12), Darrow and Thornless Evergreen blackberries (*n* = 6); Detvan, Rovada, Heros, and Jonkheer van Tets red currants (*n* = 6); Ben Lomond and Titania blackcurrants (*n* = 3); and Coville, Blue Gold, and Brigitta highbush blueberries (*n* = 3). Statistical analysis was based on two-way variance analysis with the use of Tukey’s test (*p* = 0.05). A lack of statistically significant differences between the examined groups was indicated by labeling with the same letters. A standard error (SE) was given for each mean value reported in the tables.

## 3. Results

### The Content of Anthocyanins in Different Berry Fruits

**Figure 2:**
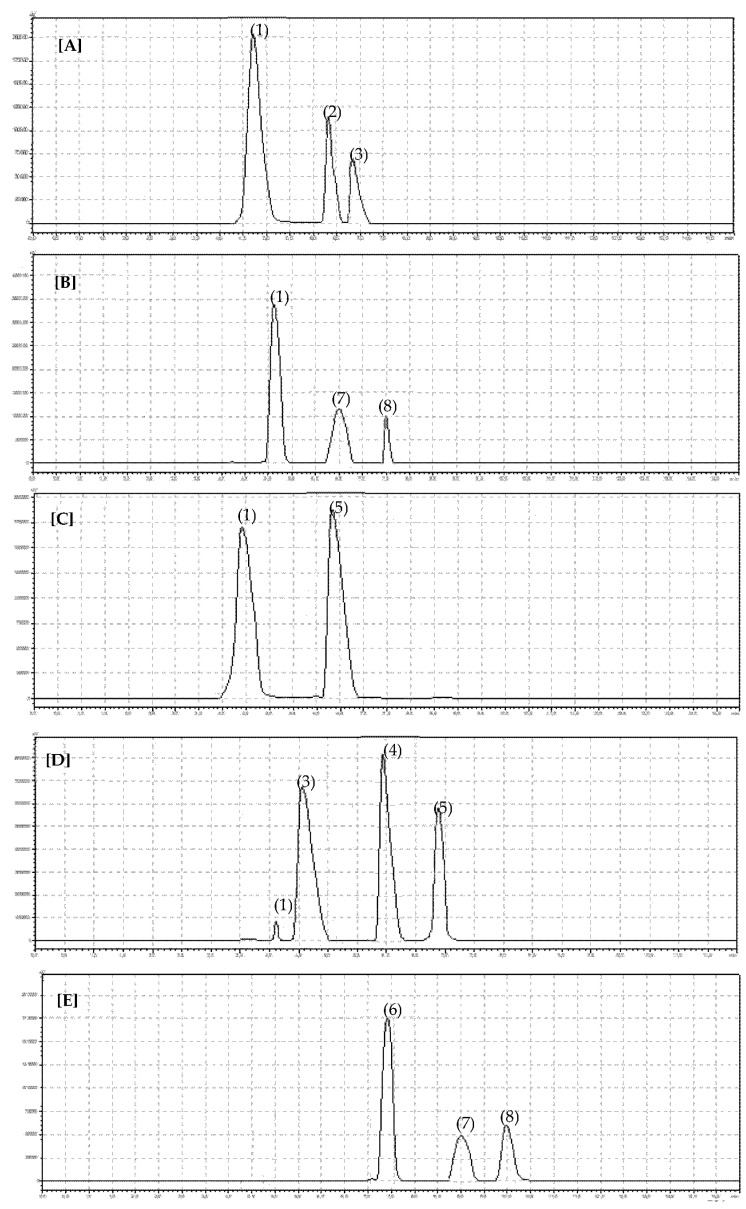
Chromatogram showing retention times for identified anthocyanins in examined berry fruits: (**A**) raspberry, (**B**) blackberry, (**C**) red currant, (**D**) blackcurrant, and (**E**) blueberry. (1) Cy-3-Glu, (2) Pel-3-Glu, (3) Del-3-Glu, (4) Del-3-Rut, (5) Cy-3-Rut, (6) Del-3-Gal, (7) Cy-3-Gal, and (8) Malv-3-Gal.

Berry-type fruits represent potent sources of anthocyanin compounds, with strong health-promoting and disease-preventive properties. The pictures of identified anthocyanis are presented in Figure 2. The analysis of selected berries commonly consumed in Poland and other counties indicated that the species and cultivar have a significant influence on the anthocyanin content of the fruit. Anthocyanins are responsible for the specific dark color of berries. It is usually believed that the darker the fruit, the more anthocyanins it contains. Our results on the concentrations of anthocyanins (total and individual for the compounds determined chromatographically) in different species and cultivars of berries are presented in Table 2 and Table 3. The results confirmed that the darkest species of berries contain the highest total anthocyanins. Among all tested species, blackberry and blackcurrant were characterized significantly (*p* < 0.0001) by the highest content of total anthocyanins (sum) (94.76 mg 100 g^−1^ f.w. and 113.79 mg 100 g^−1^ f.w.) (Table 2), while the lowest total anthocyanin content was found in red currant fruits (4.95 mg 100 g^−1^ f.w.). However, taking into account the influence of the cultivar on the total anthocyanin content in berries of different species and cultivars, the blackcurrant Titania cultivar accumulated the highest total anthocyanin content (*p* < 0.0001; 127.15 mg 100 g^−1^ f.w.) (Table 3). Blackberry was also characterized by the highest concentration of cyanidin-3-*O*-glucoside (*p* < 0.0001; 88.24 mg 100 g^−1^ f.w.) among the other examined berry species, and individual cultivars of blackberries, Darrow and Thornless Evergreen, contained, respectively, 80.98 mg 100 g^−1^ f.w. and 95.50 mg 100 g^−1^ f.w. of cyanidin-3-*O*-glucoside, whereas this anthocyanin compound was not detected in highbush blueberry.

However, in the case of pelargonidin-3-*O*-glucoside, this compound was only found in raspberry fruit (9.69 mg 100 g^−1^ f.w.). In contrast, delphinidin-3-*O*-glucoside was present in both raspberry and blackcurrant fruits, but raspberries contained significantly more of this compound (*p* = 0.0002; 34.43 mg 100 g^−1^ f.w. vs. 29. mg 100 g^−1^ f.w.). The presence of delphinidin-3-*O*-rutinoside was found only in the blackcurrant samples, and the Ben Lomond cultivar contained 51.17 mg 100 g^−1^ f.w. of this compound, while the Titania cultivar contained 69.63 mg 100 g^−1^ f.w. of delphinidin-3-*O*-rutinoside; therefore, it was also concluded that the Titania cultivar is characterized significantly by a higher content of this compound (*p* < 0.0001). Cyanidin-3-*O*-rutinoside was detected in blackberry, red currant, and blackcurrant but not in raspberry and highbush blueberry. The blackcurrant species contained the most of this compound (*p* < 0.0001; 17.48 mg 100 g^−1^ f.w.), and the Ben Lomond cultivar contained 15.64 mg 100 g^−1^ f.w of cyanidin-3-*O*-rutinoside, while the Titatnia cultivar contained 19.33 mg 100 g^−1^ f.w. of cyanidin-3-*O*-rutinoside. In contrast, delphinidin-3-*O*-galactoside and malvidin-3-*O*-galactoside were detected only in the highbush blueberry fruit samples, and the other tested species did not contain these two compounds. Highbush blueberry contained an average 48.33 mg 100 g^−1^ f.w. of malvinidin-3-*O*-galactoside and 6.45 mg 100 g^−1^ f.w. of delphinidin-3-*O*-galactoside. Among all the examined highbush blueberry cultivars, the Coville cultivar contained the most malvinidin-3-*O*-galactoside (*p* = 0.0001; 50.83 mg 100 g^−1^ f.w.), while the Brigitta cultivar contained the most delphinidin-3-*O*-galactoside (*p* = 0.0002; 7.43 mg 100 g^−1^ f.w.). However, only blackberries and highbush blueberries were distinguished by the concentration of cyanidin-3-*O*-galactoside. Moreover, the highbush blueberry fruit contained significantly more of this compound than the blackberry fruit (*p* < 0.0001; 24.77 mg 100 g^−1^ f.w. vs. 0.40 mg 100 g^−1^ f.w.). Comparing the cyanidin-3-*O*-galactoside content in the fruits of different cultivars of highbush blueberry, it was found that the fruits of the Brigitta cultivar contain the most of this compound (*p* < 0.0001; 28.53 mg 100 g^−1^ f.w.).

**Figure 3:**
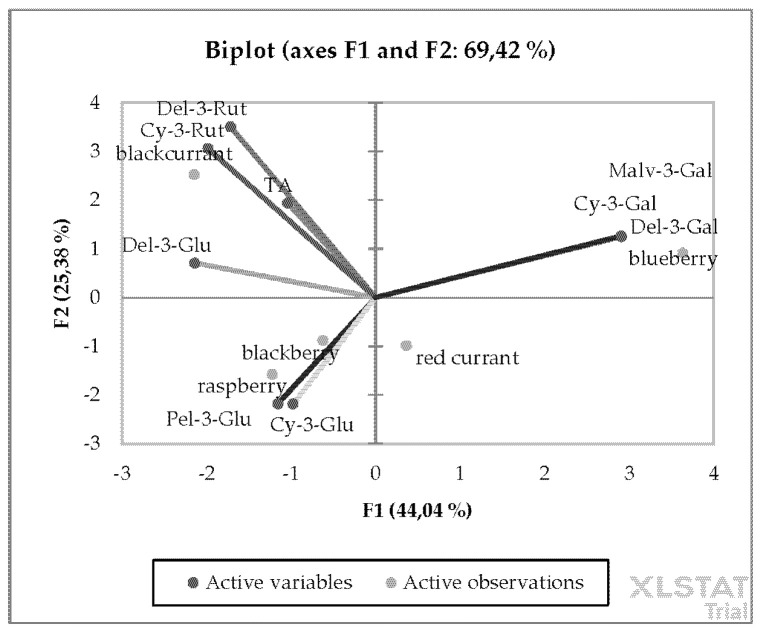
PCA showing the relationship between the anthocyanin content of different berry fruit species: total anthocyanins (TA), cyanidin-3-*O*-glucoside (Cy-3-Glu), pelargonidin-3-*O*-glucoside (Pel-3-Glu), delphinidin-3-*O*-glucoside (Del-3-Glu), delphinidin-3-*O*-rutinoside (Del-3-Rut), cyaidin-3-*O*-rutinoside (Cy-3-Rut), delphinidin-3-*O*-galactoside (Del-3-Gal), cyanidin-3-*O*-galactoside (Cy-3-Gal), and malvidin-3-*O*-galactoside (Malv-3-Gal).

PCA results showed that the overall degree of variability explained by PC1 and PC2 is 94.8% (Figure 3). This was confirmed by a strong link between the measured and identified anthocyanins and the examined berry fruits species. It is worth to point out that different berry fruits species are located in separate parts of the graph. Total anthocyanins as well mostly rutinoside derivates were more characterized in blackcurrant. In the case of galactoside derivates, we found a strong correlation with blueberry. In the last group, we found anthocyanins glucosides mostly characterized in raspberry and blackberry.

**Figure 4:**
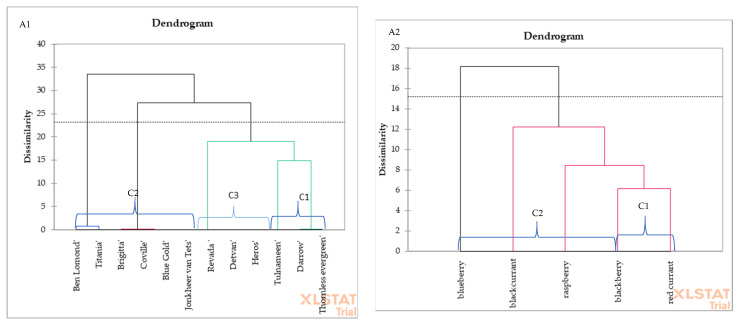
Cluster dendrograms for the berry fruit species (**A2**) (raspberry (*Rubus ideus* L.), blackberry (*Rubus* L.), red currant (*Ribes* L.), blackcurrant (*Ribes nigrum* L.), highbush blueberry (*Vaccinium corymbosum* L.)) or cultivars (**A1**) (raspberry (*Rubus ideus* L. Tulnameen cv.), blackberry (*Rubus* L. Darrow and Thornless Evergreen cv.), red currant (*Ribes* L. Detvan, Rovada, Heros, and Jonkheer van Tets cv.), blackcurrant (*Ribes nigrum* L. Ben Lomond and Titania cv.), highbush blueberry (*Vaccinium corymbosum* L., Coville, Blue Gold, and Brigitta cv.)).

The dendrogram received from hierarchical cluster analysis is shown in Figure 4. Figure 4 shows the level of genetic similarity of the berry fruit species (A2) or cultivars (A1). The higher the scale value for cultivars or species, the greater the variety between them. In terms of the content of anthocyanin compounds, blackberry and red currant form a group that can be described as homogeneous. The same applies to varieties similar to each other: blackcurrant Ben Lomond and Titania cv. are similar; likewise highbush blueberry Coville and Brigitta cv. are even more similar.

The hierarchical cluster analysis was performed to group berry fruit samples from different species or cultivars. The clustering of sample types regarding the composition of anthocyanin compounds grouped berry fruit species into two different groups (C1 and C2) and berry fruit cultivars into three different groups (C1, C2, and C3). Therefore, this result shows that the two clusters in the case of the species and the three clusters in the case of the cultivar have different chemical compositions in terms of anthocyanin compounds. Cluster 1 (Figure 4A1) demonstrates that blackberry and red currant samples were grouped closely due to contents of anthocyanins, whereas raspberry, blackcurrant, and highbush blueberry formed cluster 2 (C2) in the dendrogram (Figure 4A1). However, as can be seen in the Figure 4A2 dendrogram, Tulameen, Darrow, and Thornless evergreen cv. were grouped closely (C1); Ben Lomond, Titania, Brigitta, Coville, Blue Gold, and Jonkeer van Teets cv. formed cluster 2 (C2) in the dendrogram; and Revada, Detvan, and Heros cv. samples were grouped into cluster 3 (C3) due to similar chemical composition in terms of the content of anthocyanins.

## 4. Discussion

As mentioned in the introductory section, anthocyanins belong to the flavonoids, a class of compounds with strong antioxidant properties. The past few decades have seen a steady increase in consumer interest in all fruit species rich in bioactive compounds with antioxidant activity beneficial to health, as well as in research on these compounds [31]. Berries are of special importance among other fruits due to their unique organoleptic properties, such as color, taste, and smell; exceptionally rich nutritional value; and the possibility of various applications in gastronomy and the food industry. They can be used in fresh and processed forms in the human diet [19,32]. According to FAOSTAT statistics, the total berry production has increased gradually over the years, and the latest data for 2019 show that the world production volume of berries amounts to 922,681 metric tons. Asia and the Americas are currently the continents with the largest production of berries, followed by Europe. Among the countries, China is the largest producer of fresh berries in the world, while the EU’s top producers of berries are Spain, Germany, Italy, and Poland. Berries represent an important type of fresh produce in these countries in terms of production volume and economics [33]. At the same time, various species of berry are widespread on all continents; therefore, their importance is even greater because they are widely available and can be easily used in the diet [34]. As Poland is the leading producer of raspberries and currants in Europe, Germany of currants and gooseberries, Italy of blackberries, raspberries, and blueberries, and Spain has gained the top position in blueberry production [33], these species of berries have become the main sources of anthocyanins in the diet of Europeans. Therefore, in our research, we focused on comparing the anthocyanin content of these commonly cultivated species in Europe and some cultivars of these berries. Only 6 anthocyanins are widely spread in nature, although about 30 of these compounds have been described. Commonly occurring are pelargonidin, cyanidin, delphinidin, peonidin, petunidin, and malvinidin [19], which are the most common pigments in nature being responsible for the color of plant tissues [8].

In our study on raspberry fruits, the anthocyanin profile was predominated by cyanidin-3-glucoside, which accounted for around 51% of total anthocyanins, followed by delphinidin-3-*O*-glucoside, which accounted for 38%, and pelargonidin-3-*O*-gucoside, which accounted for 11%. Veberic and coauthors reported that this species contained the highest amounts of cyanidin glycosides (83.8%), followed by pelargonidin glucosides (16.2%), and thus, the color of the raspberry fruit is bright red. According to the authors, berry species contain two to six different cyanidin glycosides [35]. The anthocyanin profile, specific and characteristic to raspberry fruits, defined by Määttä-Riihinen et al. [36], consists of cyanidin-3-sophoroside, cyanidin-3-glucoside, cyandin-3-rutinoside, and cyanidin-3-glucoside-r-utinoside. These findings are in line with the results presented by Veberic et al. [35], who reported that cyanidin 3-sophoroside is the main cyanidin gycoside occurring in raspberries. In the study conducted by Corderio et al. [37], the anthocyanin profile of the raspberry samples was as follows: cyjanidin-3-sophoroside, cyjanidin-3(2-glucosylrutinoside), cyjanidin-3-glucoside, pelargonidin-3-sophoroside, and cyjanidin-3-rutinoside. The Tulnameen cultivar tested in our study contained 89.54 mg 100 g^−1^ f.w. of anthocyanins. Generally, this is in agreement with the previously reported data. Babinaite et al. [38] reported that among the 17 red-fruited raspberry cultivars, the anthocyanin content ranged from 29.2 to 130.6 mg 100 g^−1^ f.w. Similar results were obtained by Weber and coauthors [39] comparing 30 cultivars, who showed the contents of anthocyanin being in the range from 35 to 112 mg 100 g^−1^ f.w., expressed as cyjanidin-3-glucoside.

In blackberry fruits, we identified cyjanidin-3-*O*-glucoside as a predominant compound with a share of 93% of all anthocyanins detected, while 6.5% was cyjanidin-3-*O*-rutinoside and the rest 0.4% was cyjanidin-3-*O*-galactoside. Anthocyanins occurring in blackberry are mainly cyanidin-based anthocyanins, and the primary anthocyanin is cyanidin-3-*O*-glucoside [40]. Some authors also reported the detection of various other anthocyanins in blackberries, such as cyanidin-3-*O*-xyloside, cyanidin-3-*O*-dioxaloylglucoside, and cyanidin-3-*O*-(600-malonyl)-glucoside, as well as pelargonidin-3-*O*-glucoside, malvidin-3-*O*-glucoside, cyanidin-3-*O*-arabinoside, cyanidin-3-*O*-xyloside, cyanidin-3-*O*-rutinoside, cyanidin-3-*O*-dioxalylglucoside, and cyanidin-3-*O*-glucoside acylated with malonic acid in smaller amounts [41]. The total anthocyanin content of blackberry fruits detected in our study ranged approximately from 87 to 102 mg 100 g^−1^ f.w. This is quite similar to the range of 70.3–201 mg 100 g^−1^ f.w. and the mean of 117 mg 100 g^−1^ f.w. for 18 blackberry cultivars reported by Fan-Chiang and Wrolstad [42] but somewhat lower than Van de Velde et al.’s [40] work, who revealed the concentration of anthocyanins in the three blackberry cultivars at a level ranging from 107 mg to 124 mg in 100 g of fresh matter. Our results showed that the Tornless Evergreen cultivar produces a larger amount of anthocyanins than the thorny one, Darrow, which is consistent with Koloniak-Ostek et al.’s [0] report. In the case of the Thornless Evergreen cultivar, we detected 102.5 mg 100 g^−1^ f.w. of anthocyanins, which is similar to the content shown by Fan-Chiang and Wrolstad, who reported in the same cultivar the amount of 117 mg 100 g^−1^ f.w. [42]. According to another study, Thornless Evergreen berries contain a higher anthocyanin content expressed as cyanidin-3-glucoside equivalent than in our study (146.8 mg 100 g^−1^ f.w.) [43]. The difference may be due to a different method of anthocyanin determination. The previously mentioned authors [42] detected five anthocyanins in blackberries: cyanidin-3-glucoside, cyanidin-3-rutinoside, cyanidin-3-xyloside, cyanidin-3-glucoside acylated with malonic acid, and an unidentified acylated derivative of cyanidin-3-glucoside, while Van de Velde and coauthors detected four cyjanidin-based compounds, and cyanidin-3-*O*-glucoside was the major anthocyanin detected in all blackberry samples, representing more than 85% of the total anthocyanins. This finding is in agreement with our results and with the reports for other blackberry cultivars [42]. The papers of Jordheim et al. [44] and Veberic et al. [35], who notified that blackberries only biosynthesize cyanidin glycosides as primary metabolites, also confirm our results on the identified anthocyanins in blackcurrant fruit.

In the case of red currants, cyanidin glycosides are the only group of anthocyanins present in the berries [35]. According to our results, the sum of anthocyanins detected ranged from 4.3 to 6.2 mg 100 g^−1^ f.w., depending on the cultivar, and is lower than in Zdunic and coauthors’ research [45]. The authors reported an anthocyanin content from 7.1 to 19.3 mg 100 g^−1^ f.w. in individual cultivars of red currant. In the case of Rovada and Jonkheer van Tets cultivars, which we also analyzed, the contents were 8.2 and 18.8 mg 100 g^−1^ f.w., respectively, while we gained 4.89 and 6.20 mg 100 g^−1^ f.w. of anthocyanins (sum), respectively. In Zdunic et al.’s study [45], 8.4 mg 100 g^−1^ f.w. was cyanidin, while 5.3 mg 100 g^−1^ f.w. was delphinidin in the Rovada cultivar. At the same time, in Jonkheer van Tets, 16.0 mg 100 g^−1^ f.w. was cyanidin, while 4.3 mg 100 g^−1^ f.w. was delphinidin. Other authors [43] reported the anthocyanin content expressed as cyanidin-3-glucoside equivalents in the Rovada cultivar on the level of 7.5 mg 100 g^−1^ f.w. In our study, in all three cultivars of red currants tested, we detected only two anthocyanin compounds, cyanidin-3-*O*-rutinoside and cyanidin-3-*O*-glucoside, and the first one accounted for about 60%. Borges et al. [46] also reported that the main anthocyanins in the red currant extract were cyanidin-3-*O*- rutinoside and cyanidin-3-*O*-rutinoside.

In our study, delphinidins (delphinidin-3-*O*-glucoside and delphinidin-3-*O*-rutinoside) were dominating anthocyanins in blackcurrants, constituting 76.1% and 80.5% of the sum of anthocyanins detected, respectively, depending on the cultivar. This corresponds to other authors’ reports on the anthocyanin profile of blackcurrants. According to Veberic et al. [35], delphinidin has the highest share (60.7%) in blackcurrant anthocyanins, followed by cyanidin (30.6%) and peonidin (0.9%). Other authors have reported the highest share of delphinidin-3-*O*-rutinoside and cyanidin-3-*O*-rutinoside, followed by delphinidin-3-*O*-glucoside in blackcurrants [47]. This result was in accordance with other studied references [45,48,49,50]. Laczko-Zold et al. [51] in their study detected as the prevailing anthocyanin cyanidin-3-*O*-glucoside (82.7% share) in blackcurrant fruits. The mean value of the sum of four anthocyanins detected in two blackcurrant cultivars was 113.79 mg 100 g^−1^ f.w., which is in agreement with that reported in the literature [48,52], but some authors have reported a higher total anthocyanin content expressed as milligrams of cyanidin-3-glucoside per 100 g of f.w. in blackcurrants [53]. In our study, two cultivars Titania and Ben Lomond were tested, and the higher anthocyanin content was found in the Titania cultivar (127.15 mg 100 g^−1^ f.w.) in comparison to the other one (100.43 mg 100 g^−1^ f.w.). These results are partly confirmed by studies of other authors. Wojdyło et al. [48] reported a similar anthocyanin content in the Titania cultivar, but Benvenuti et al. [53] revealed twofold more anthocyanins in the Ben Lomond cultivar.

Blueberries are referred to as a fruit that guarantees longevity due to the fact that they are considered one of the greatest sources of antioxidants of all fruits and vegetables [31]. They belong to berries with a high content of anthocyanins, which is confirmed by many studies included in the review by Gündeşli et al., according to which the content of these compounds in the fruit ranges from 57 to 503 mg 100 g^−1^ f.w. [31]. These results are in alignment with our findings and suggest varietal differences in the anthocyanin content of blueberry fruits. The other studies [54,55,56,57] confirmed that the values resulting from the quantitative analysis of total anthocyanins in blueberries are in a quite wide range and are strongly dependent on the blueberry cultivar and on the method used for analysis. Additionally, as Skrovankova et al. [57] underline, the phenolics, in that the anthocyanin content of blueberries, are influenced by the degree of maturity at harvest, as well as growing practices and growing locations. During blueberry ripening, polyphenols are transformed toward the synthesis of anthocyanins, which may confirm the above statements. Another reason for the big differences in the anthocyanin content of blueberries may also be the size of the fruit. Anthocyanins occur mostly in the blueberry skin, so the small-sized fruits have a relatively higher skin area and, thus, a higher anthocyanin content [58]. Compositional analysis of the anthocyanins in seven cultivars of blueberries [55] showed that malvinidin is the major anthocyanin component of fruits (representing 30–47% of the total anthocyanins). Stevenson and Scalzo [58] in their review also summarized that the predominating anthocyanin in blueberries is malvidin, followed by delphinidin, cyanidin, petunidin, and pelargonidin in similar quantities. In our study, the most abundant anthocyanins were malvinidin-3-*O*-galactoside (60.8%) and cyanidin-3-*O*-galactoside (31.1%), while delphinidin-3-*O*-galactoside, on average, constituted 8.1% of anthocyanins in three cultivars tested. The long-term evaluation of the anthocyanin composition of blueberries belonging to over 80 cultivars showed a significant variability related to the vegetation seasons and between cultivars [58]. We found a similar content of anthocyanins in the three tested cultivars: Coville, Blue Gold, and Brigitta (from approx. 76 to 84 mg 100 g^−1^ f.w.), which was quite consistent with the amount of these compounds in the same cultivars included in the works of Stevenson and Scalzo, which were 100, 206, and 101.8 mg of 100 g^−1^ f.w., respectively [58].

The described observations on the level of the content of detected anthocyanins in the berry fruits and PCA allow us to conclude that individual compounds are closely correlated with the species of berries. Dark-colored blackcurrants contain mainly delphinidin-3-*O*-rutinoside and cyanidin-3-*O*-rutinoside, while red currants, which have much brighter fruits than blackcurrant, have the lowest content of anthocyanins of all the berries tested. On the other hand, for botanically related blackberries and raspberries, the most characteristic are the same compounds such cyanidin-3-*O*-glucoside and pelargonidin-3-*O*-glucoside. In addition, in the case of blueberries, there is a clear correlation with anthocyanin compounds such as delphinidin-3-*O*-galactoside, cyanidin-3-*O*-galactoside, and malvidin-3-*O*-galactoside, which are not found in other berries in such high amounts. These results have also been confirmed by other authors in studies of anthocyanin components in fruits [37,42,46,52,59]. According to the literature, fruit, berries, and red wine are most frequently reported as contributing foods to dietary anthocyanin intake, which varies depending on the country, season, and dietary habits [34,59]. In contrast, berries of numerous species and cultivars eaten by consumers contain anthocyanins in far greater quantities than other food representatives such as wine [59]. To summarize our findings, the average amount of anthocyanins in berry fruits investigated is in the following increasing order: red currant < blueberry < raspberry, red < blackberry < blackcurrant. This indicates that last three mentioned berries are significant sources of anthocyanins, which is consistent with findings of other authors cited above. The differences between different berry species in the content of anthocyanins are closely associated with the color of their skin. Berries with red, blue, or purple colors belong to one of the most important sources of anthocyanins, which occur mainly in the external layers of the skin, while flesh tissues (in vacuoles in the form of granules) contain much less or no anthocyanins [35]. This is the explanation that light-colored fruits like red currants have less anthocyanins in their composition. Additionally, some of the above-described differences in anthocyanin content and composition within individual berry species are due to the fact that the chemical composition of plants is influenced by various factors like cultivar and tissue, cultivation method, climatic conditions of the growing area, as well as ripening stage of berries and storage conditions [36,46,49,53]. The mentioned discrepancies indicate that the quantitative and qualitative profiles of anthocyanins occurring in berries may vary significantly depending on these factors. This can also be related to the method of anthocyanin detection, as the works cited sometimes differed in this respect.

## 5. Conclusions

The study showed that the species and cultivars of berry fruits differ significantly in the content of anthocyanin compounds. The darkest species of berries contain the highest total anthocyanins. Among all the tested samples, blackberry and blackcurrant were significantly characterized by the highest total anthocyanin content, with the lowest being in red currant fruit. Additionally, the content of individual anthocyanin compounds in particular species and cultivars also varied. Although different species and cultivars of berry fruits show a high variability in anthocyanin profiles, they all should be recognized as a rich source of bioactive compounds with pro-health potential. This study expands our knowledge about the variation in the content of valuable anthocyanins in berry fruits and may help in the selection and validation of the most productive species. More work is needed on identifying, quantifying, and decoding anthocyanins in fruits of different berry species and cultivars, in that in the skin, flesh, and seeds of these berries.

## Figures and Tables

**Table 1:** LOD and LOQ values for all anthocyanins used in the presented experiment (mg/100 g f.w.).

Compound	LOD	LOQ
Cyanidin-3-*O*-glucoside	0.4	1.2
Cyanidin-3-*O*-rutinoside	0.8	2.4
Cyanidin-3-*O*-galactoside	0.1	0.2
Delphinidin-3-*O*-glucoside	2.2	6.7
Delphinidin-3-*O*-rutinoside	1.0	3.0
Delphinidin-3-*O*-galactoside	1.0	3.0
Pelargonidin-3-*O*-glucoside	0.5	1.5
Malvidin-3-*O*-galactoside	0.8	2.4

**Table 2:** The average content of individual anthocyanins in different species of berry fruits (in mg 100 g^−1^ f.w.). Mean value ± SE.

Species	TA	Cy-3-Glu	Pel-3-Glu	Del-3-Glu	Del-3-Rut	Cy-3-Rut	Del-3-Gal	Cy-3-Gal	Malv-3-Gal
Raspberry (*Rubus ideus* L.)	89.54 ± 3.02ab	45.42 ± 1.54b	9.69 ± 0.45	34.43 ± 1.17a	<LOD	<LOD	<LOD	<LOD	<LOD
Blackberry (*Rubus* L.)	94.76 ± 3.16a	88.24 ± 2.97a	<LOD	<LOD	<LOD	6.12 ± 0.22b	<LOD	0.40 ± 0.02b	<LOD
Red currant (*Ribes* L.)	4.95 ± 0.24c	1.94 ± 0.09c	<LOD	<LOD	<LOD	3.00 ± 0.16b	<LOD	<LOD	<LOD
Blackcurrant (*Ribes nigrum* L.)	113.79 ± 5.46a	6.54 ± 0.43c	<LOD	29.36 ± 1.34b	60.40 ± 3.75	17.48 ± 0.76a	<LOD	<LOD	<LOD
Highbush blueberry (*Vaccinium corymbosum* L.)	79.55 ± 1.18b	<LOD	<LOD	<LOD	<LOD	<LOD	6.45 ± 0.28	24.77 ± 0.93a	48.33 ± 0.69
*p*-Value (species)	<0.0001	<0.0001		0.0002		<0.0001		<0.0001	

Total anthocyanins (TA) (sum); cyanidin-3-*O*-glucoside (Cy-3-Glu); pelargonidin-3-*O*-glucoside (Pel-3-Glu); delphinidin-3-*O*-glucoside (Del-3-Glu); delphinidin-3-*O*-rutinoside (Del-3-Rut); cyanidin-3-*O*-rutinoside (Cy-3-Rut); delphinidin-3-*O*-galactoside (Del-3-Gal); cyanidin-3-*O*-galactoside (Cy-3-Gal); malvidin-3-*O*-galactoside (Malv-3-Gal); limit of detection LOD. Means in the same column followed by the same letter are not significantly different (*p* < 0.05) by Tukey’s test.

**Table 3:** The content of individual anthocyanins in different cultivars of berry fruits (in mg 100 g^−1^ f.w.). Mean value ± SE.

Species	Cultivar	TA	Cy-3-Glu	Pel-3-Glu	Del-3-Glu	Del-3-Rut	Cy-3-Rut	Del-3-Gal	Cy-3-Gal	Malv-3-Gal
Raspberry (*Rubus ideus* L.)	Tulnameen	89.54 ± 3.02bcd	45.42 ± 1.54b	9.69 ± 0.45a	34.43 ± 1.17a	<LOD	<LOD	<LOD	<LOD	<LOD
Blackberry (*Rubus* L.)	Darrow	87.02 ± 0.37bcde	80.98 ± 0.25a	<LOD	<LOD	<LOD	5.70 ± 0.18c	<LOD	0.35 ± 0.01d	<LOD
	Thornless Evergreen	102.50 ± 0.09b	95.50 ± 0.12a	<LOD	<LOD	<LOD	6.55 ± 0.18c	<LOD	0.45 ± 0.02d	<LOD
Red currant (*Ribes* L.)	Detvan	4.39 ± 0.32e	1.73 ± 0.08c	<LOD	<LOD	<LOD	2.66 ± 0.24d	<LOD	<LOD	<LOD
	Rovada	4.89 ± 0.40e	1.92 ± 0.13c	<LOD	<LOD	<LOD	2.97 ± 0.2d	<LOD	<LOD	<LOD
	Heros	4.30 ± 0.1 e	1.60 ± 0.01c	<LOD	<LOD	<LOD	2.70 ± 0.14d	<LOD	<LOD	<LOD
	Jonkheer van Tets	6.20 ± 0.26e	2.52 ± 0.13c	<LOD	<LOD	<LOD	3.68 ± 0.13b	<LOD	<LOD	<LOD
Blackcurrant (*Ribes nigrum* L.)	Ben Lomond	100.43 ± 0.39bc	7.54 ± 0.23c	<LOD	26.07 ± 0.10b	51.17 ± 0.39b	15.64 ± 0.18a	<LOD	<LOD	<LOD
	Titania	127.15 ± 0.43a	5.55 ± 0.15c	<LOD	32.64 ± 0.01ab	69.63 ± 0.25a	19.33 ± 0.03a	<LOD	<LOD	<LOD
Highbush blueberry (*Vaccinium corymbosum* L.)	Coville	78.25 ± 0.48de	<LOD	<LOD	<LOD	<LOD	<LOD	5.41 ± 0.12c	22.02 ± 0.36c	50.83 ± 0.16a
	Blue Gold	76.14 ± 0.62d	<LOD	<LOD	<LOD	<LOD	<LOD	6.52 ± 0.12b	23.75 ± 0.06b	45.87 ± 0.44c
	Brigitta	84.25 ± 0.16cde	<LOD	<LOD	<LOD	<LOD	<LOD	7.43 ± 0.11a	28.53 ± 0.07a	48.29 ± 0.07b
*p*-Value (cultivar)		<0.0001	<0.0001	<0.0001	0.0099	<0.0001	<0.0001	0.0002	<0.0001	0.0001

Total anthocyanins (TA) (sum); cyanidin-3-*O*-glucoside (Cy-3-Glu); pelargonidin-3-*O*-glucoside (Pel-3-Glu); delphinidin-3-*O*-glucoside (Del-3-Glu); delphinidin-3-*O*-rutinoside (Del-3-Rut); cyanidin-3-*O*-rutinoside (Cy-3-Rut); delphinidin-3-*O*-galactoside (Del-3-Gal); cyanidin-3-*O*-galactoside (Cy-3-Gal); malvidin-3-*O*-galactoside (Malv-3-Gal); limit of detection LOD. Means in the same column followed by the same letter are not significantly different (*p* < 0.05) by Tukey’s test.

## Data Availability

Data will be made available upon reasonable request by authors Alicja Ponder and Ewelina Hallmann.
